# Supplementary material for: Infants Display Anticipatory Gaze During a Motor Contingency Paradigm
Source: Sensors (Basel). 2025 Jan 30;25(3):844. doi: 10.3390/s25030844 (PMC11820326; doi:10.3390/s25030844)
Supplement: Supplementary file 1 [file sensors-25-00844-s001.zip › sensors-3396860-supplementary.pdf]

Supplemental video S1. Recording of the ROI for Figure 3a and Figure 3b. The ROI for each region is boxed in yellow.

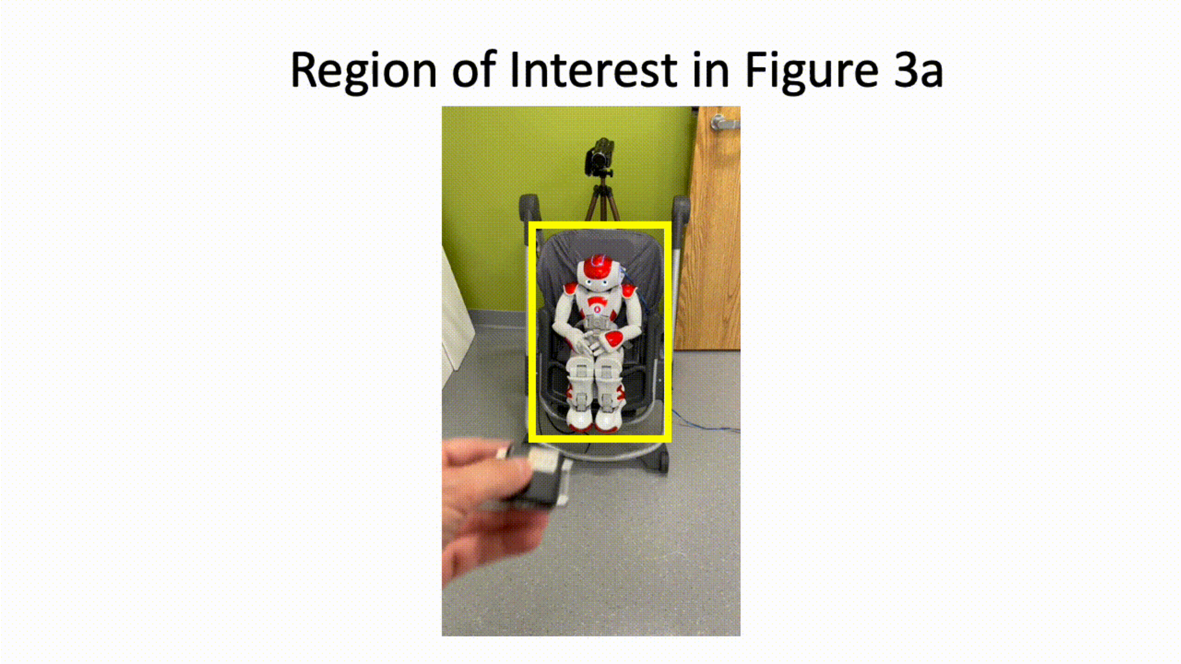

Supplemental Figure S1. Individual plot for each infant, from each learning classification. The title above each graph denotes the infant ID number, learning classification, and their total number of activations (L= Classically defined learner, NL- Classically defined non-learner). Graph a) is a bar graph that plots the total number of activations on the y-axis and the minute blocks of the contingency period on x-axis. Graph b) is a line graph that plots the frequency of the type of gaze in the y axis for the same minute blocks described in graph a. The colors denote the types of gazes (blue = non-robot looks, red = reactive looks, yellow = predictive looks).

Classically Defined Learners

L1- 100 activations

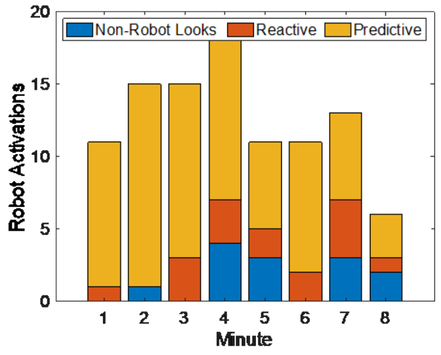

a)

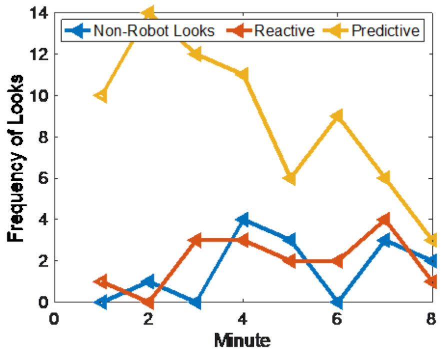

b)

L2- 63 activations

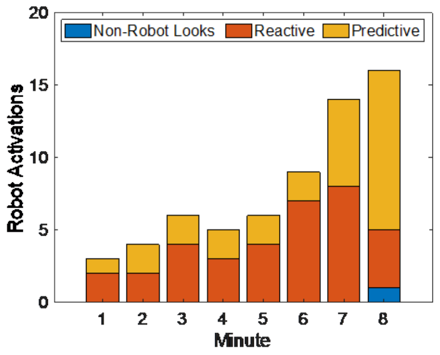

a)

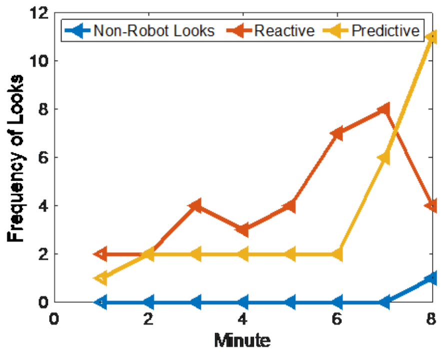

b)

L3 - 66 activations

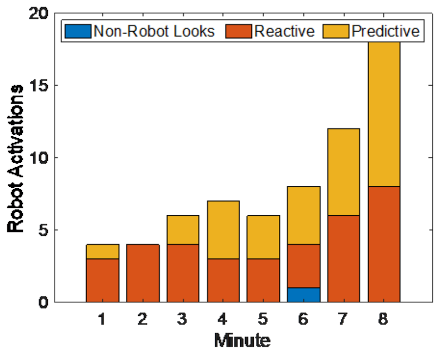

a)

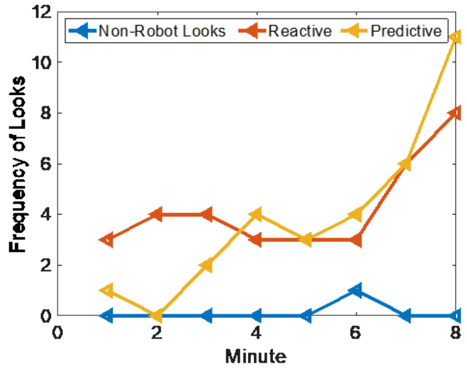

b)

L4 - 87 activations

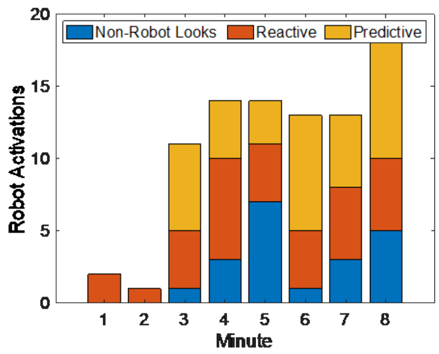

a)

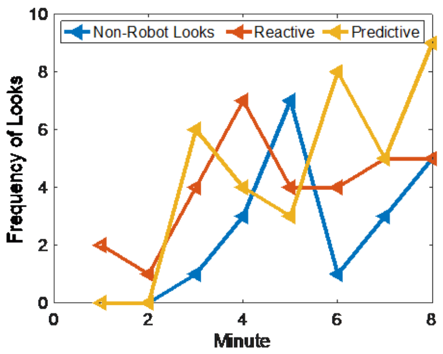

b)

L5 - 77 activations

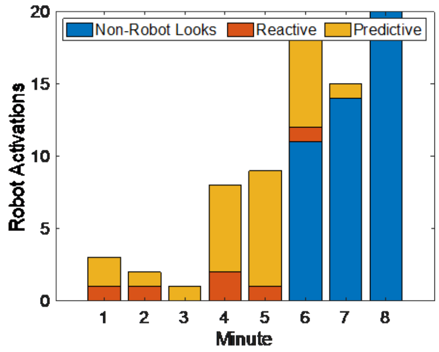

a)

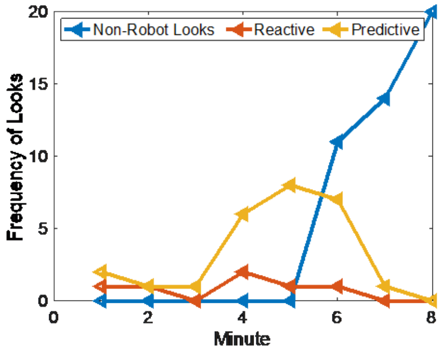

b)

L6 - 41 activations

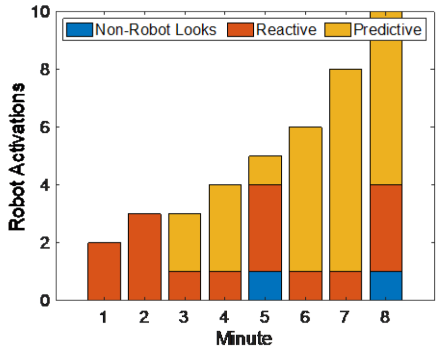

a)

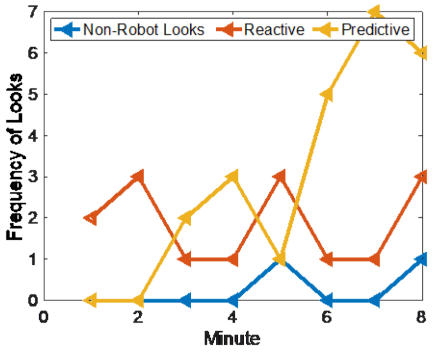

b)

Classically Defined Non-Learners

NL1- 90 activations

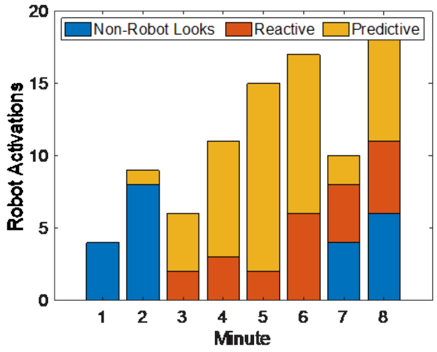

a)

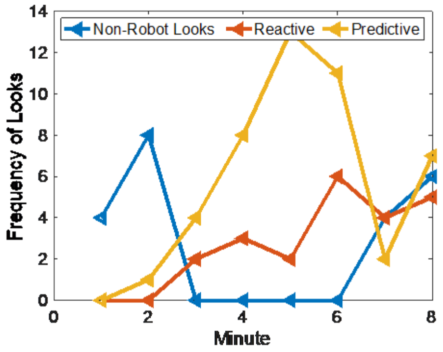

b)

NL2 - 124 activations

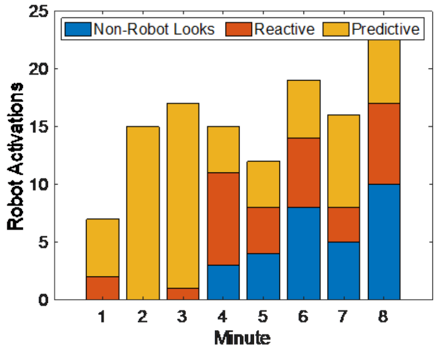

a)

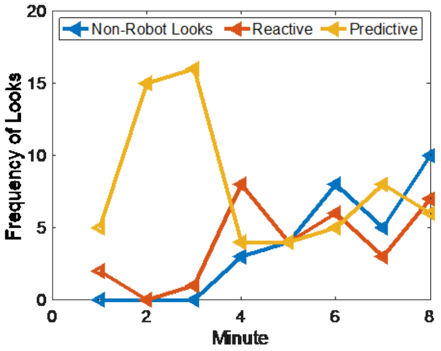

b)

NL5 - 43 activations

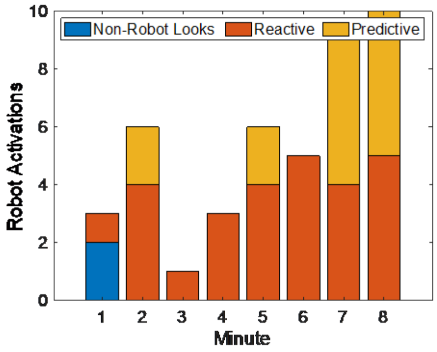

a)

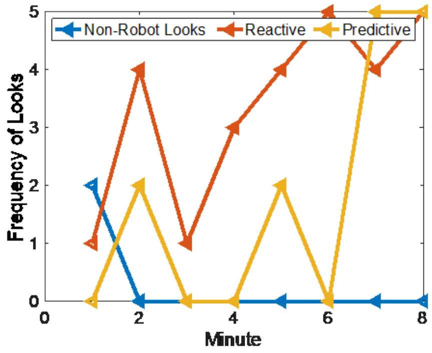

b)

NL6 - 110 activations

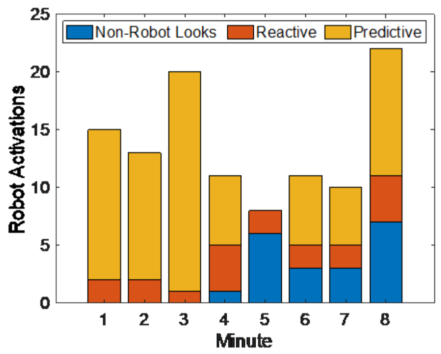

a)

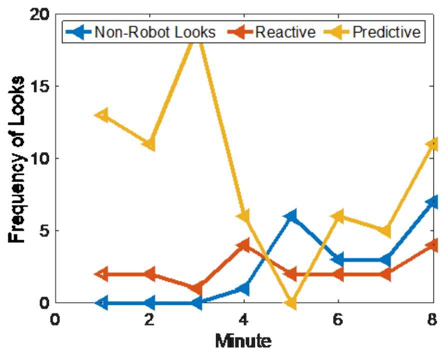

b)

NL7 - 33 activations

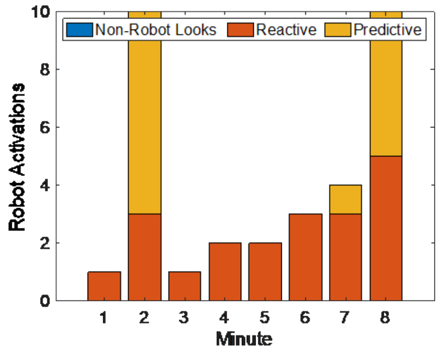

a)

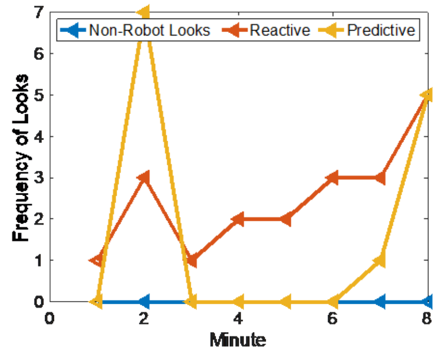

b)

NL8 - 72 activations

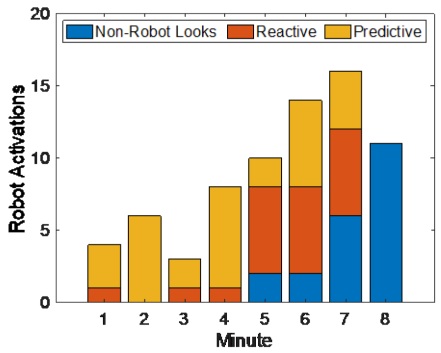

a)

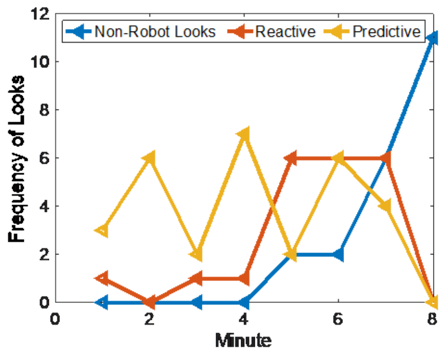

b)

NL9 - 97 activations

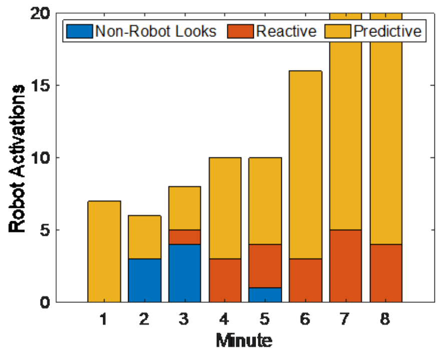

a)

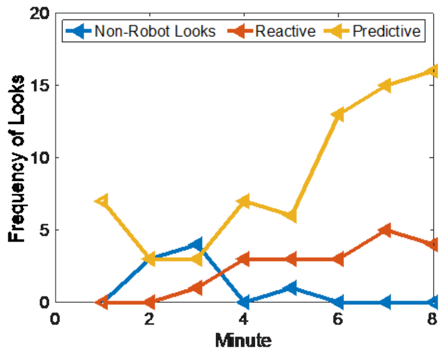

b)

Supplement Figure S2. Individual bar plot for each infant depicting the amount of time spent looking (y-axis) during each minute block (x-axis) of the contingency. The infant's ID number is displayed to the left of the infant's graph. The classification of learning is denoted as L for learners and NL for non-learners.

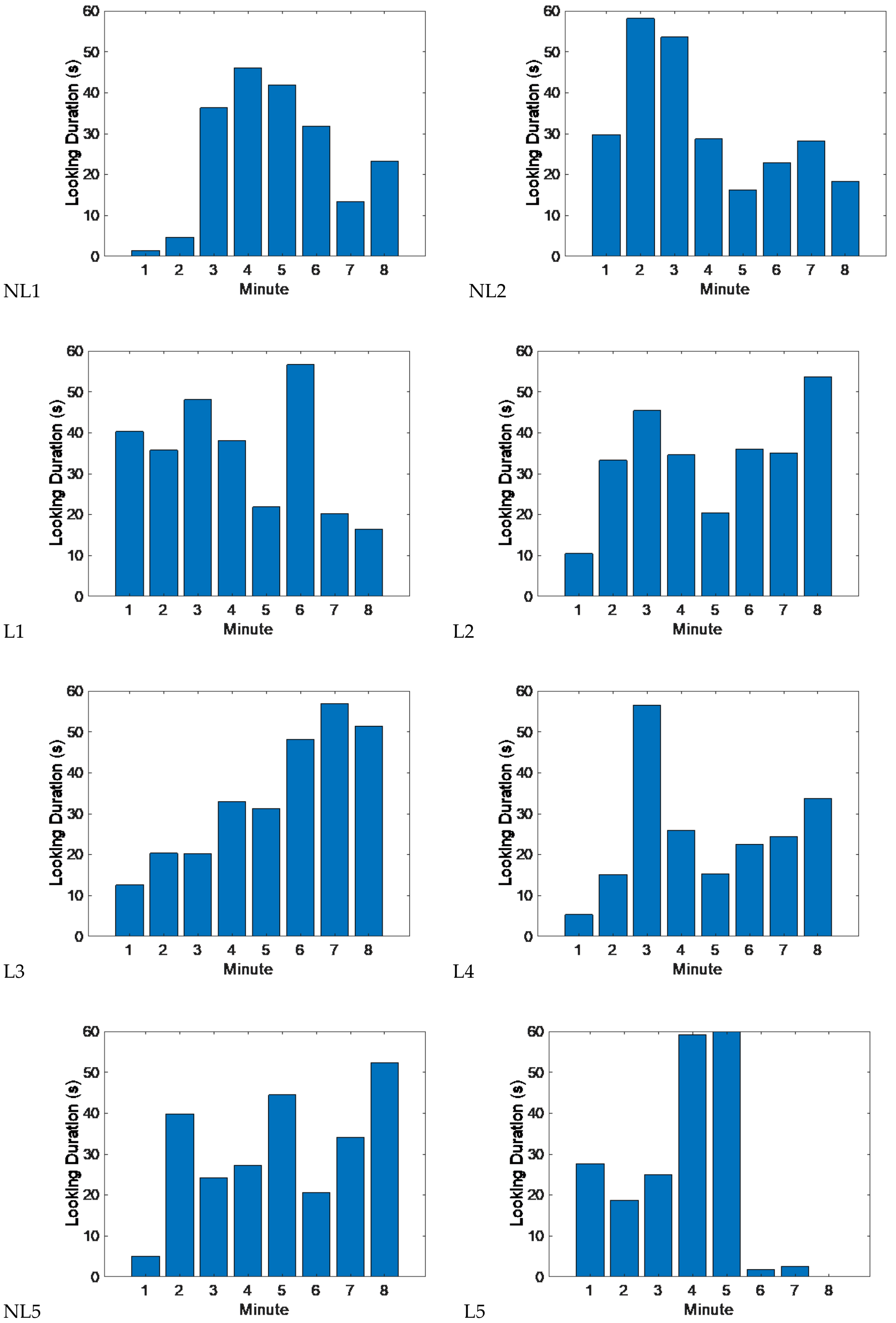

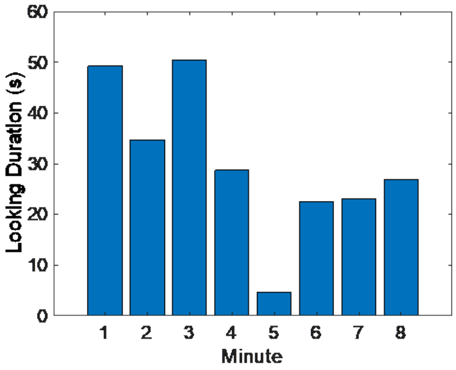

NL6

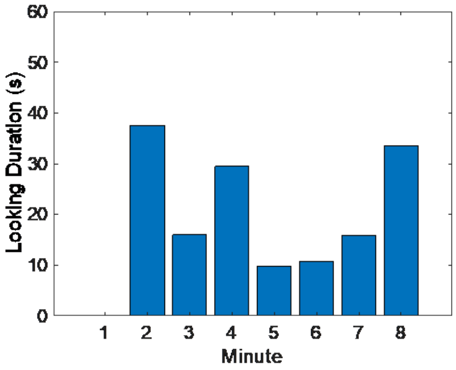

NL7

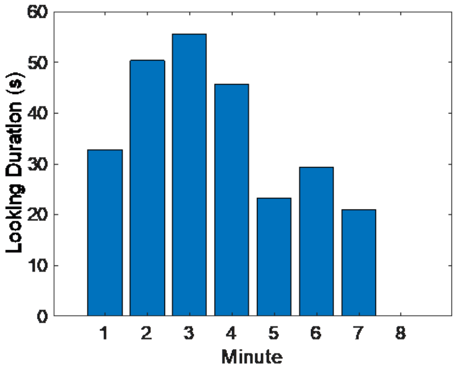

NL8

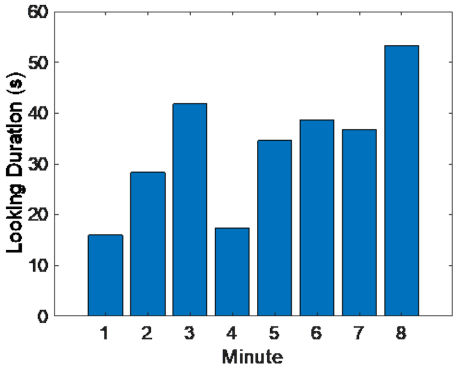

L6

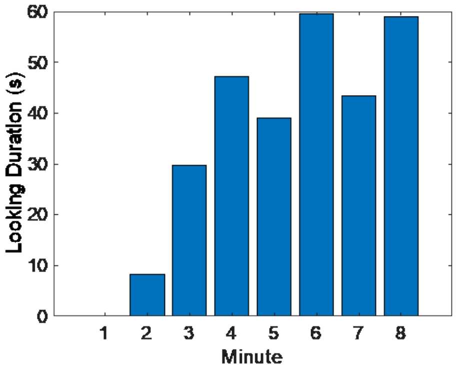

NL9
